# P3H4 is correlated with clinicopathological features and prognosis in bladder cancer

**DOI:** 10.1186/s12957-018-1507-2

**Published:** 2018-10-15

**Authors:** Wangjian Li, Lihong Ye, Yongliang Chen, Peng Chen

**Affiliations:** 1 Department of Urology, Shaoxing Hospital of China Medical University, Zhejiang, China; 2 Department of Radiation Oncology, Hangzhou Cancer Hospital, Zhejiang, China

**Keywords:** Bladder cancer, P3H4, Clinicopathological features, Prognosis

## Abstract

**Background:**

Genetic alterations play a significant role in the progression of bladder cancer. Identifying novel biomarkers to personalize the therapeutic regimen and evaluate the prognosis of patients with bladder cancer is vital. Prolyl 3-hydroxylase family member 4 (P3H4) is significantly involved in several types of human cancer. However, the effect of P3H4 in bladder cancer remains unknown.

**Methods:**

The mRNA expression of P3H4 was measured in 44 paired tumors and adjacent normal tissues by using real-time reverse transcription-polymerase chain reaction. RNA-Seq data of 389 patients with bladder cancer were downloaded to investigate the effect of P3H4 on bladder cancer from The Cancer Genome Atlas (TCGA) project.

**Results:**

P3H4 was overexpressed in bladder cancer compared with the adjacent normal tissue both in our tissue samples and TCGA samples. The mRNA expression of P3H4 was significantly related to several clinicopathological factors of bladder cancer, including age, race category, histologic grade, tumor histologic subtype, and AJCC stage. The high P3H4 expression group had a shorter overall survival (OS) than the low P3H4 expression group. Univariate Cox regression analysis showed that age, angiolymphatic invasion, lymph node metastasis, tumor histologic subtype, metastasis, AJCC stage, and P3H4 were significantly related to OS. Moreover, multivariate Cox analysis revealed that P3H4, as well as age and AJCC stage, was an independent predictor of poor OS.

**Conclusion:**

Given its tumorigenic role, P3H4 may serve as a promising tumor-promoting gene in bladder cancer.

**Electronic supplementary material:**

The online version of this article (10.1186/s12957-018-1507-2) contains supplementary material, which is available to authorized users.

## Background

Bladder cancer is a common cancer worldwide. An estimated 429,800 new cases of bladder cancer and 165,100 deaths were recorded in 2012 [1]. Bladder cancer is also the leading malignancy of the genitourinary system [2]. The median survival of patients with advanced bladder cancer is only about 14 months even after aggressive treatment, including surgery, radiation, and chemotherapy [3].

Cancer progression is a complex process that involves changes in various genes, including oncogenes, tumor suppressor genes, and non-coding sequence. Genetic profiling studies have indicated that many genetic alterations, such as those in TP53, RB1, TSC1, FGFR3, and PIK3CA, play critical roles in bladder cancer [4–10]. Genetic studies have significantly progressed recently, but many areas remain to be explored.

Prolyl 3-hydroxylase family member 4 (P3H4) is a nucleolar protein initially identified as an autoantigen in cases of interstitial cystitis [11]. P3H4 may also play an important role in membranous nephropathy [12]. Another study indicated that P3H4 is a novel endoplasmic reticulum protein that regulates bone mass homeostasis [13]. P3H4 could affect the activity of lysyl-hydroxylase 1 potentially through interactions with the enzyme and/or cyclophilin B [14]. Fosså et al. reported that P3H4 acts as a tumor-associated autoantigen in patients with prostate cancer [15]. Comtesse et al. found that patients with meningioma possess antibodies against P3H4 and may offer a new diagnostic and therapeutic target for meningioma [16]. Overall, P3H4 is involved in several physiological and pathological processes. Nevertheless, the role of P3H4 in bladder cancer remains unknown.

The present study analyzed the relationship between P3H4 and bladder cancer. In specific, the relationship between P3H4 and clinicopathological factors of patients with bladder cancer was mainly investigated. The prognostic value of P3H4 was also assessed by Kaplan–Meier and Cox regression analyses.

## Methods

### Patients

Forty-four bladder cancer and corresponding adjacent normal tissues were obtained from Shaoxing Hospital of China Medical University. Tissue samples were first frozen in liquid nitrogen after resection and stored in a − 80 °C refrigerator until use. The research protocol was approved by the Ethics Committee of Shaoxing Hospital of China Medical University. Moreover, written informed consent for using the tissue samples was obtained from those 44 patients. All samples are muscle invasive bladder cancer (MIBC).

### RNA extraction and quantitative real-time polymerase chain reaction

RNA of the tissue samples was extracted using TRIzol reagent in accordance with the manufacturer’s instructions (Life Technologies, USA). Quantitative real-time polymerase chain reaction (qRT-PCR) was carried out using Thunderbird SYBR qPCR Mix (Toyobo, Japan) in the Applied Biosystems 7500 Real-Time PCR System (Applied Biosystems, USA). The primers for P3H4 were ACGCGCTGTTCAAGGCTAA (forward) and CCAGCATCCCCTGATAGTAGT (reverse). GAPDH was used as an internal control.

### TCGA clinical and P3H4 data

We downloaded TCGA clinical data in “Biotab” format from The Cancer Genome Atlas. Then, the normalized mRNA expression counts of P3H4 were acquired from TCGA and are expressed as RNA-Seq by transcripts per kilobase million values.

### Statistical method

Data on normal distribution were expressed as mean ± standard deviation and were then compared with *t* test. Data on abnormal distribution were compared with Mann–Whitney *U* test. Chi-square test or Fisher’s exact test was used to evaluate the connection between clinicopathological characteristics and P3H4 expression, as appropriate. Kaplan–Meier and Cox regression analyses were performed to assess the prognostic value of P3H4. Statistical significance was considered at *p* < 0.05. All statistical analyses were carried out using SPSS, and GraphPad Prism 5 was used for drafting.

## Results

### P3H4 was significantly upregulated in bladder cancer

**Fig. 1:**
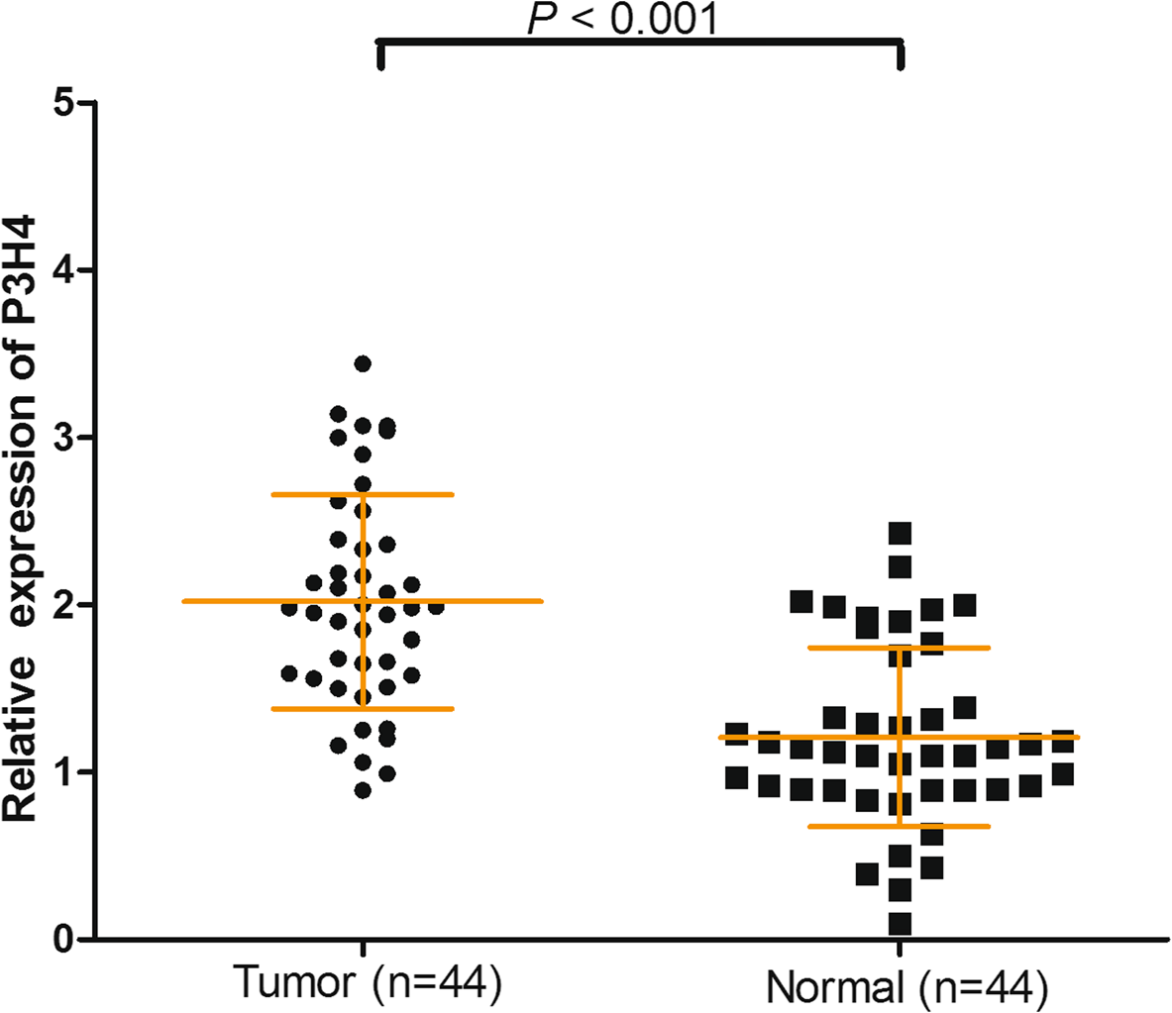
P3H4 expression in 44 paired bladder cancer samples and adjacent normal tissues was investigated using qRT-PCR. P3H4 expression was significantly upregulated in primary bladder cancer tissues compared with the adjacent normal tissues (*p* < 0.001)

**Fig. 2:**
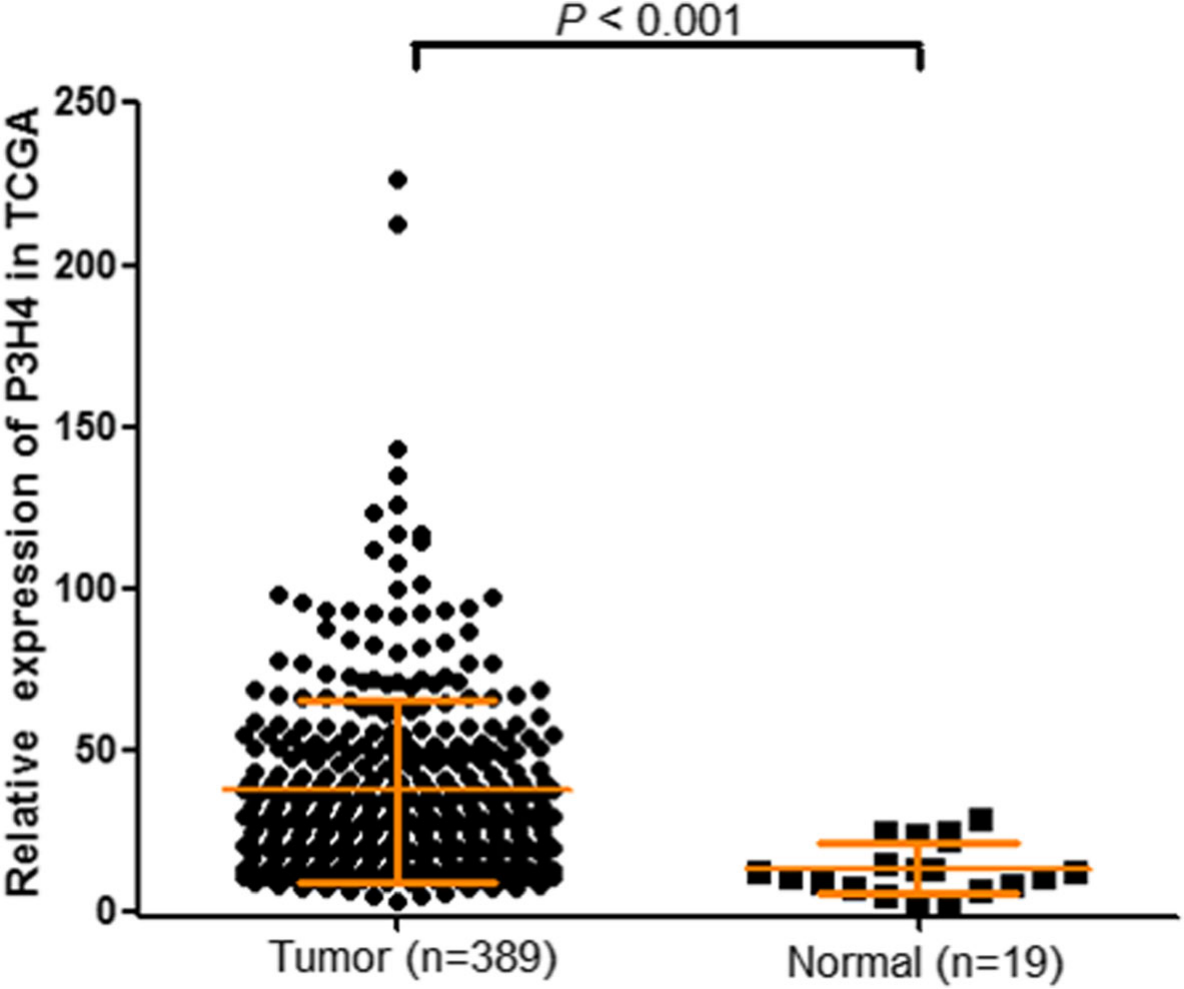
P3H4 expression in the TCGA cohort, including 389 bladder cancer samples and 19 adjacent normal samples. P3H4 expression was also significantly overexpressed in primary bladder cancer tissue (*p* < 0.001)

To examine the role of P3H4 in bladder cancer, we investigated the P3H4 expression in 44 primary tumors and their paired adjacent normal tissues using qRT-PCR. P3H4 was upregulated in primary tumor compared with their paired adjacent normal tissues (2.02 ± 0.64 vs 1.21 ± 0.54, *p* < 0.001, Fig. 1). To further confirm the results, we analyzed the expression of P3H4 in 389 bladder cancer tissues and 19 adjacent normal tissues from the TCGA database. As shown in Fig. 2, the expression of P3H4 was also significantly upregulated in the TCGA database (37.77 ± 28.27 vs 13.60 ± 7.67, *p* < 0.001). This finding revealed that P3H4 may play a significant role in bladder cancer.

### P3H4 expression was associated with clinicopathological features in bladder cancer

To further explore whether P3H4 expression is related to clinicopathological factors of bladder cancer, we divided 389 patients with bladder cancer from the TCGA database into high P3H4 expression (*n* = 195) and low P3H4 expression (*n* = 194) groups according to the median value of P3H4. The results from the TCGA cohort indicated that P3H4 expression was significantly associated with age, race category, histologic grade, tumor histologic subtype, and AJCC stage (*p* < 0.05, Table 1). The average age was greater in the high P3H4 expression group than in the low P3H4 expression group (69.25 ± 10.18 vs 66.39 ± 10.87, *p* = 0.008). Similar results were also found in our local cohort (71.55 ± 7.71 vs 66.32 ± 7.92, *p* = 0.032, Additional file 1: Table S1). Interestingly, the race category was also different between the two groups (*p* = 0.001). The high P3H4 expression group had a higher histologic grade and more non-papillary type percentage than the low P3H4 expression group (98.45% vs 90.63% and 73.82% vs 61.66%, *p* = 0.001 and 0.011, respectively). The most significant finding is that high P3H4 expression corresponded to advanced AJCC stage (*p* = 0.005). Gender, BMI, angiolymphatic invasion, extracapsular extension, Karnofsky performance score, lymph node metastasis, and metastasis showed no significant correlation with P3H4 expression (*p* > 0.05). Overall, high P3H4 expression may play an important role in bladder cancer.

**Table 1:** The relationship between P3H4 expression and clinicopathological characteristics in the TCGA cohort (*n* = 389)

Characteristics	Expression of P3H4 mRNA, number (%)		
	High (*n* = 195)	Low (*n* = 194)	*p* value
Age at diagnosis, years	69.25 ± 10.18	66.39 ± 10.87	**0.008**
Gender			0.053
Male	136 (69.74)	152 (78.35)	
Female	59 (30.26)	42 (21.65)	
BMI	26.71 ± 4.78	27.42 ± 6.99	0.277
Race category			**0.001**
White	163 (87.63)	145 (77.54)	
Black or African American	13 (6.99)	9 (4.81)	
Asian	10 (5.38)	33 (17.65)	
Angiolymphatic invasion			0.181
Yes	77 (57.04)	64 (48.85)	
No	58 (42.96)	67 (51.15)	
Extracapsular extension			0.937
Yes	35 (44.30)	31 (43.66)	
NO	44 (55.70)	40 (56.34)	
Karnofsky performance score	84.33 ± 12.46	80.67 ± 15.39	0.146
Histologic grade			**0.001**
High	191 (98.45)	174 (90.63)	
Low	3 (1.55)	18 (9.38)	
Lymph node metastasis			0.454
Yes	59 (38.31)	53 (42.74)	
No	95 (61.69)	71 (57.26)	
Tumor histologic subtype			**0.011**
Non-papillary	141 (73.82)	119 (61.66)	
Papillary	50 (26.18)	74 (38.34)	
Metastasis			0.154
M0	79 (91.86)	110 (97.35)	
M1	7 (8.14)	3 (2.65)	
AJCC stage			**0.006**
I	0 (0)	1 (0.52)	
II	49 (25.13)	79 (41.15)	
III	75 (38.46)	55 (28.65)	
IV	71 (36.41)	57 (29.69)	

Boldface means *p* < 0.05

### P3H4 expression was significantly related to prognosis in bladder cancer

**Fig. 3:**
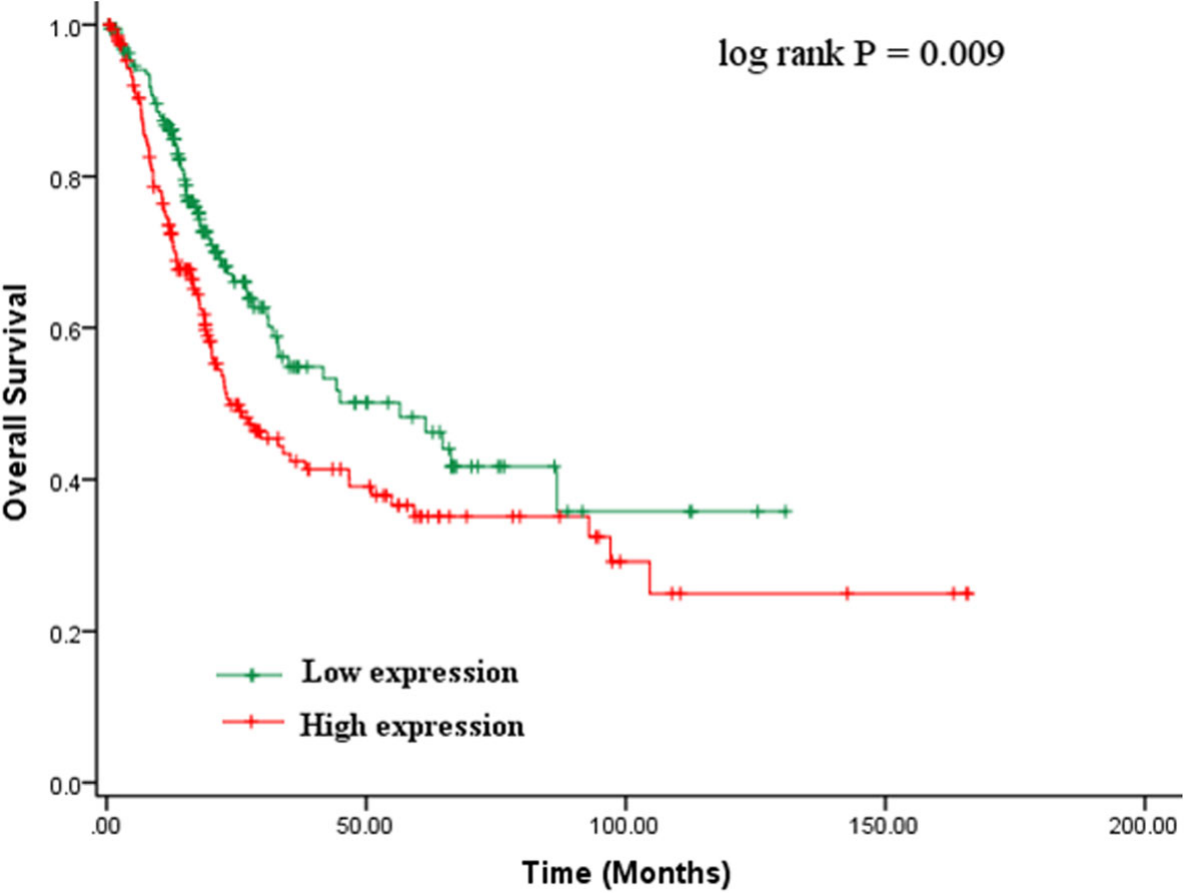
Kaplan–Meier analysis for the overall survival of patients with bladder cancer in the TCGA cohort. The high P3H4 expression group had a shorter overall survival than the low P3H4 expression group (*p* = 0.009)

To further investigate the prognostic role of P3H4 in bladder cancer, we performed Kaplan–Meier and Cox regression analyses. Kaplan–Meier analysis showed that survival prognosis was different between the high and low P3H4 expression groups. The high P3H4 expression group had a shorter overall survival (OS) than the low P3H4 expression group (*p* = 0.009, Fig. 3). The cumulative 5-year OS in the high P3H4 expression group was 35.1%, which was much lower than that in the low P3H4 expression group (48.3%). Univariate and multivariate Cox regression analyses were then performed. Univariate Cox regression analysis showed that age (hazard ratio (HR) = 1.04, 95% CI = 1.023–1.057, *p* < 0.001), angiolymphatic invasion (HR = 1.619, 95% CI = 1.177–2.226, *p* = 0.003), lymph node metastasis (HR = 2.071, 95% CI = 1.47–2.918, *p* < 0.001), tumor histologic subtype (HR = 0.628, 95% CI = 0.433–0.909, *p* = 0.014), metastasis (HR = 4.507, 95% CI = 2.131–9.532, *p* < 0.001), AJCC stage (HR = 1.708, 95% CI = 1.402–2.08, *p* < 0.001), and P3H4 (HR = 1.504, 95% CI = 1.104–2.048, *p* = 0.01) were associated with OS (Table 2). However, other factors, including gender, BMI, race category, extracapsular extension, Karnofsky performance score, and histologic grade, were not significantly related to OS (*p* > 0.05, Table 2). Moreover, multivariate Cox analysis indicated that only age (HR = 1.037, 95% CI = 1.019–1.054, *p* < 0.001), AJCC stage (HR = 1.560, 95% CI = 1.265–1.923, *p* < 0.001), and P3H4 (HR = 1.413, 95% CI = 1.016–1.966, *p* = 0.04) were independent factors of poor OS in bladder cancer (Table 3). Taken together, the results indicate that P3H4 is a poor independent prognostic factor of bladder cancer and worthy of further study.

**Table 2:** Univariate Cox regression analysis of P3H4 expression with regard to OS

Clinicopathologic features	HR	95% CI	*p* value
Age	1.04	1.023–1.057	**< 0.001**
Gender	1.08	0.771–1.513	0.655
BMI	0.994	0.965–1.023	0.683
Race category	1.158	0.862–1.555	0.33
Angiolymphatic invasion	1.619	1.177–2.226	**0.003**
Extracapsular extension	1.359	0.936–1.974	0.107
Karnofsky performance score	0.998	0.976–1.021	0.893
Histologic grade	2.853	0.705–11.542	0.141
Lymph node metastasis	2.071	1.47–2.918	**< 0.001**
Tumor histologic subtype	0.628	0.433–0.909	**0.014**
Metastasis	4.507	2.131–9.532	**< 0.001**
AJCC stage	1.708	1.402–2.08	**< 0.001**
P3H4	1.504	1.104–2.048	**0.01**

Boldface means *p* < 0.05

**Table 3:** Multivariate Cox regression analysis of P3H4 expression with regard to OS

Clinicopathologic features	HR	95% CI	*p* value
Age	1.037	1.019–1.054	**< 0.001**
AJCC stage	1.560	1.265–1.923	**< 0.001**
P3H4	1.413	1.016–1.966	**0.04**

Boldface means *p* < 0.05

## Discussion

Bladder cancer is the second most common urological cancer worldwide and the most frequent urological cancer in China [17]. Surgical operation is the major treatment for bladder cancer. Despite the recent significant progress in surgical techniques and adjuvant chemotherapy, bladder cancer remains a highly fatal disease [18, 19]. The recurrence rate of this disease after local treatment is high [20]. Owing to the limited prediction abilities of conventional markers, such as clinicopathological characteristics, identifying reliable genetic markers that predict disease progression is vital [21, 22].

In the present study, we studied the association between P3H4 gene expression and bladder cancer. P3H4 is a nucleolar protein initially identified as an autoantigen in interstitial cystitis cases [11]. Previous studies have found that P3H4 may play an important role in several human diseases, such as membranous nephropathy [12], bone mass homeostasis [13], and Ehlers–Danlos syndrome [23]. Moreover, P3H4 is significantly associated with several types of human cancer. However, the relationship between P3H4 and bladder cancer remains unclear to date.

In the present study, we reported and validated that P3H4 was significantly upregulated in primary tumor compared with their paired adjacent normal tissues in 44 patients with bladder cancer. The differential expression of P3H4 was further confirmed in the TCGA cohort of a large sample size. Furthermore, high P3H4 expression was found to be closely related to several aggressive clinicopathological factors, such as advanced AJCC stage. Moreover, Kaplan–Meier analysis found that the low P3H4 expression group had a better prognosis than the high P3H4 expression group. Univariate and multivariate Cox regression analyses revealed that P3H4 was an independent predictor of poor OS in bladder cancer. Taken together, our findings suggest that P3H4 is involved in the progression of bladder cancer. A systematic literature review revealed that this was the first description of the relationship between P3H4 and bladder cancer.

Despite some encouraging findings, several limitations of our study cannot be ignored. First, although it had been verified by small samples, the relationship between P3H4 expression and bladder cancer should be validated using a large sample data from our local cohort. Second, future studies should conduct in vitro and in vivo experiments to identify the biological roles of P3H4 in bladder cancer. The molecular mechanism of P3H4 in bladder cancer remains to be further studied.

## Conclusions

We identified P3H4 as a tumor promotion gene in bladder cancer for the first time. P3H4 was overexpressed in primary tumor tissues compared with their paired adjacent normal tissues. Moreover, high P3H4 expression was associated with aggressive clinicopathological features in bladder cancer. Survival analysis revealed that high P3H4 expression correlated poorly with prognosis in bladder cancer. These results indicate that P3H4 may act as a tumor-promoting gene in bladder cancer.

## Additional files

Additional file 1: **Table S1.**The relationship between P3H4 expression and clinicopathological characteristics in our local cohort (*n* = 44). (DOCX 18 kb)

## Abbreviations

HR: Hazard ratio
OS: Overall survival
P3H4: Prolyl 3-hydroxylase family member 4
qRT-PCR: Quantitative real-time polymerase chain reaction
TCGA: The Cancer Genome Atlas
