# Supplementary material for: P3H4 is correlated with clinicopathological features and prognosis in bladder cancer
Source: World J Surg Oncol. 2018 Oct 15;16:206. doi: 10.1186/s12957-018-1507-2 (PMC6190559; doi:10.1186/s12957-018-1507-2)
Supplement: Supplementary file 1 — Table S1.The relationship between P3H4 expression and clinicopathological characteristics in our local cohort (n = 44). (DOCX 18 kb) [file 12957_2018_1507_MOESM1_ESM.docx]

| Table S1. The relationship between P3H4 expression and clinicopathological characteristics in our local cohort (n=44) | | | |
| --- | --- | --- | --- |
| Characteristics | Expression of P3H4 mRNA, Number (%) | | |
|  | HIGH(n=22) | LOW(n=22) | P-value |
| Age at diagnosis, y | 71.55±7.71 | 66.32±7.92 | **0.032** |
| Gender |  |  | 0.15 |
| Male | 15 | 19 |  |
| Female | 7 | 3 |  |
| Angiolymphatic Invasion |  |  | 0.741 |
| Yes | 7 | 6 |  |
| No | 15 | 16 |  |
| Histologic Grade |  |  | 0.600 |
| High | 21 | 19 |  |
| Low  Pathological subtype  Nonurothelial  Urothelial  Tumor Stage  T2  T3  T4 | 1  3  19  5  12  4 | 3  2  20  6  11  5 | 1.000  0.895 |
| Lymph Node Metastasis |  |  |  |
| Yes | 6 | 6 |  |
| No | 16 | 16 |  |
| Metastasis |  |  |  |
| M0 | 22 | 22 |  |
| M1 | 0 | 0 |  |
| AJCC stage |  |  | 0.578 |
| I  II  III | 0  4  11 | 0  7  9 |  |
| IV | 7 | 6 |  |
